# Silver-based SERS substrates fabricated using a 3D printed microfluidic device

**DOI:** 10.3762/bjnano.14.65

**Published:** 2023-07-21

**Authors:** Phommachith Sonexai, Minh Van Nguyen, Bui The Huy, Yong-Ill Lee

**Affiliations:** 1 Anastro Laboratory, Institute of Basic Science, Changwon National University, Changwon 51140, Republic of Korea; 2 Department of Pharmacy, Pharmaceutical Technical University, Tashkent 100084, Uzbekistan

**Keywords:** 3D printing, microfluidic droplet, SERS substrate, silver nanoparticle, smartphone detection

## Abstract

The detection of harmful chemicals in the environment and for food safety is a crucial requirement. While traditional techniques such as GC–MS and HPLC provide high sensitivity, they are expensive, time-consuming, and require skilled labor. Surface-enhanced Raman spectroscopy (SERS) is a powerful analytical tool for detecting ultralow concentrations of chemical compounds and biomolecules. We present a reproducible method for producing Ag nanoparticles that can be used to create highly sensitive SERS substrates. A microfluidic device was employed to confine the precursor reagents within the droplets, resulting in Ag nanoparticles of uniform shape and size. The study investigates the effects of various synthesis conditions on the size distribution, dispersity, and localized surface plasmon resonance wavelength of the Ag nanoparticles. To create the SERS substrate, the as-synthesized Ag nanoparticles were assembled into a monolayer on a liquid/air interface and deposited onto a porous silicon array prepared through a metal-assisted chemical etching approach. By using the developed microfluidic device, enhancement factors of the Raman signal for rhodamine B (at 10^−9^ M) and melamine (at 10^−7^ M) of 8.59 × 10^6^ and 8.21 × 10^3^, respectively, were obtained. The detection limits for rhodamine B and melamine were estimated to be 1.94 × 10^−10^ M and 2.8 × 10^−8^ M with relative standard deviation values of 3.4% and 4.6%, respectively. The developed SERS substrate exhibits exceptional analytical performance and has the potential to be a valuable analytical tool for monitoring environmental contaminants.

## Introduction

Surface-enhanced Raman spectroscopy (SERS) has emerged as a powerful optical trace detection technique in various biochemical applications because of its exceptional sensitivity and the capabilities of real-time analysis and label-free detection [1–2]. SERS has been used to identify targets for single molecules in chemical and biological systems [3–4] since its discovery by Martin Fleischmann in 1974 [5]. Electromagnetic and chemical mechanisms are attributed to the SERS enhancement. In electromagnetic theory, the excitation of metal particles through light leads to localized surface plasmon resonance due to the collective oscillation of free electrons in the confined space of the metal particles. The electric field is enhanced, and the Raman enhancement factor (EF) can reach 10^6^ [6]. The induced amplification of the local field by plasmonic coupling occurs in nanometer-scale regions around the metal particles, the so-called electromagnetic “hot spots”. The chemical mechanism suggests the formation of a charge-transfer complex between chemisorbed species and matrix material, which yields enhancement when the excitation frequency resonates with a charge-transfer transition [7].

Noble metal nanoparticles (NPs) have gained much popularity in various fields, such as analytical chemistry and catalytic chemistry, where they have been used to develop localized surface plasmon resonance (LSPR) and SERS substrates [8]. For example, Ag NPs yield a strong SERS effect at relatively low cost. However, an issue often encountered in synthetic approaches is the non-uniformity of the Ag NPs. Homogeneous Ag NPs are necessary for Ag-based SERS substrates to function well. The microfluidic approach is a technique for the fine control and manipulation of fluids, in which capillary penetration is limited to the micrometer scale and mass transport dominates [9–10]. Microfluidic devices are used in various fields in industry and laboratories, such as chemical synthesis and microreactors [11–12], drug screening [13], and clinical trials [14]. They can create homogeneous reaction environments with controllable parameters for synthesizing homogeneous colloidal nanoparticles with a narrow size distribution [15–17]. Two types of microfluidic devices are commonly used, namely droplet-based and continuous devices. Continuous microfluidic devices have several disadvantages when applied for chemical syntheses, such as laminar flow formation due to the low Reynolds number of fluids, which leads to a lack of uniform mixing [18]. In addition, nanoparticles tend to accumulate on the sidewalls of the microfluidic channel, thus limiting the reuse of the device [8,19]. In contrast, droplet-based microfluidic devices are based on the formation of microdroplets using two or more immiscible fluids with reactants in segmented flow and oil in continuous flow. They can control the reaction rates by mass transfer through convection and diffusion [20–21]. Furthermore, droplet-based microfluidic devices are frequently utilized to synthesize complex materials of uniform size, as each droplet can function as a separate microreactor [12,22].

A traditional approach to producing microfluidic devices involves a three-step microfabrication process of (i) creating a channel mold using photolithography, (ii) fabricating the channels by casting the mold through soft lithography, and (iii) bonding the channel device to a substrate [23]. This procedure typically requires a clean room and expensive facilities such as a photolithography machine, a spin-coater, and photoresist agents, as well as long processing times and well-trained technical staff. Additionally, the photolithography process is limited to planar fabrication, resulting in a low aspect ratio of the achieved features.

Because 3D printing enables the creation and testing of objects in short periods of time, it provides a new tool for constructing microfluidic devices. This has led to fast and dynamic developments in chemical synthesis and analytical systems at low cost [24–26]. There are two techniques for producing 3D-printed microfluidic devices. In the first approach, monolithic microfluidic devices are 3D printed [27–28]. Although this one-step process offers the benefits of quick development and ease of fabrication, reducing the channel dimension to a scale smaller than a millimeter remains challenging [29]. In the second approach, 3D printing can replace photolithography to fabricate a mold. This approach can achieve a better lateral resolution of printed features down to 100 µm with a higher aspect ratio of the printed channel features [30]; also, it does not require a clean room. The stereolithography (SLA) technique is an additive manufacturing technique in which a photopolymer resin is cured and converted from a liquid to a solid by an ultraviolet laser. The resolution of SLA printers is determined by the radial beam scattering and the type of resin [31]. With particular resins, SLA can fabricate features with lateral dimensions of 100 µm and a mold-printed resolution of 50 µm.

Over the past decade, numerous SERS substrates based on various materials, including paper [32–33], polymers [34–35], fibers [36], dielectrics [37], porous aluminum oxide [38], and semiconductors [39] have been reported. Dielectric and semiconductor substrates, such as ZnO nanowires, silicon nanowires, and porous silicon (PS), are particularly popular because of their larger contribution to the amplification of the Raman signal and longer shelf life [40–42]. Silicon nanostructures with high specific areas are especially popular because they have no fluorescence properties.

In this work, we report on the synthesis of a highly sensitive SERS substrate for detecting rhodamine B (RhB) and melamine (MLM) and on the analytical properties of the developed system. The substrate was prepared from Ag NPs by decorating the surface of porous silicon with Ag NPs using self-assembled monolayers (SAMs). The SAM method offers precise control, versatility, simplicity, stability, and compatibility, making it a valuable technique for surface modification in various scientific and technological applications. The Ag NPs were synthesized using a droplet-based microfluidic device and a stereolithographic 3D printing method. The microfluidic device was optimized to produce uniform droplets, within which silver nitrate was reduced by sodium borohydride. This method limits the amount of precursor chemicals and enables the sequential flow of droplets, resulting in silver nanoparticles of uniform shape and size. We investigated the effects of different synthesis conditions on the size distribution, dispersity, and LSPR wavelength of the silver nanoparticles.

## Experimental

### Chemicals and apparatus

Silver nitrate (AgNO_3_, 99.9%) was purchased from Kojima Chemical (Japan). Sodium borohydride (NaBH_4_, 98%) and melamine (99%) were obtained from Sigma-Aldrich (Republic of Korea). Hydrofluoric acid (48–51%), sulfuric acid (98%), nitric acid (65–70%), rhodamine B (pure), mineral oil (light molecular biology grade), sorbitan monooleate (SPAN 80), and trisodium citrate dehydrate (TCD) (99%) were purchased from Alfa Aesar (Republic of Korea). Sylgard 184 A&B was obtained from Sewang Hitech (Republic of Korea), and the XL-1500 UV cross-linker used for UV curing was purchased from Krackeler Scientific (USA). Methyl alcohol, hydrogen peroxide (35%), sodium hydroxide (<97%), ammonia water (25–29%), acetone (99.5%), ethyl alcohol (95%), and 2-propanol (99.5%) were bought from Daejung (Republic of Korea). The Formlabs 3D printer and clear V4 resin were purchased from Formlabs (USA). Deionized water with a resistivity of 18 MΩ·cm^−1^, provided by a Milli-Q water purification system (Millipore Corp., MA, USA), was used throughout all the experiments.

The morphologies of Ag NPs and SERS substrates were examined using a field-emission scanning electron microscope (FE-SEM, Mira II, TESCAN). A UV–vis spectrometer (Agilent 8453, Agilent, USA) and a micro-Raman spectrometer (NS200, Nanoscope System, Republic of Korea) were used to record the absorption spectra and Raman spectra, respectively.

### Fabrication of the droplet-based microfluidic device

The fabrication process of the microfluidic device is illustrated in Scheme S1 (Supporting Information File 1). The 3D model of the microfluidic device mold was designed using Solidworks Professional 2022 SP3.1 software and printed on a Formslab 3 SLA 3D printer (Formlabs Inc., Somerville, MA, USA) using Clear V4 resin. The mold was post-treated by being soaked for 20 min in a 90% (v/v) isopropanol solution, followed by 10 min in deionized water, and then gently dried in a nitrogen flow. After that, the mold was exposed to UV light at an energy level of 120 mJ·cm^−2^ for 30 s and then annealed at 60 °C for 12 h in an oven for slow evaporative drying (Scheme S1a, Supporting Information File 1). Before casting into the printed mold, polydimethylsiloxane (PDMS) Sylgard^TM^ 184 and curing agent (10:1, w/w) were mixed for 10 min and de-bubbled in vacuum for 30 min. The PDMS cast was dried for 18 h to cure after being dried in air for 24 h (Scheme S1b, Supporting Information File 1). After that, the cast was peeled off from the mold and was immersed for 24 h in 1 M NaOH solution (Scheme S1c, Supporting Information File 1). Finally, the cast was carefully adhered to the surface of a glass plate (Scheme S1d, Supporting Information File 1) and treated at 90 °C for 5 min on a hot plate. Following this step, silicon tubes were joined to the inlets of the microfluidic device using Loctite superglue.

### Synthesis of silver nanoparticles

The droplet-based microfluidic device was used to synthesize Ag NPs. These droplets enable the uniform distribution of nanoparticles. Ag NPs were created by reducing AgNO_3_ solution with NaBH_4_ as a reducing agent and TCD as a stabilizer [43–44]. Mineral oil served as a continuous phase when combined with the surfactant Span 80 (2% w/v). To prevent droplets from coalescing in the microchannel, Span 80 was added to lower the interfacial tension between oil and aqueous phase.

For the synthesis of Ag NPs, 20 mL of AgNO_3_ and 20 mL of TCD were mixed in a 1:3 molar ratio to form the reactant solution, designated as solution (a). 20 mL of NaBH_4_ and NaOH were mixed in a 1:3 molar ratio, designated as solution (b). Two syringe pumps (NE-300 InfusionONE Syginge Pump, USA) were used to inject mineral oil mixed with the surfactant Span 80 (2% w/v), solution (a), and solution (b) into the droplet-based microfluidic system at flow rates of 20 and 80 µL·min^−1^, respectively.

The as-synthesized colloidal solution of Ag NPs was centrifuged at 12,000 rpm for 5 min to separate the Ag NPs solution and remove the oil phase. Finally, the Ag NPs suspension was stored in a dark vial at room temperature for further experiments.

### Fabrication of porous silicon (PS) substrate

A p-type Si(100) wafer with a resistivity of 0.01–0.09 Ω·cm was used in this work. The wafer was divided into 1 × 1 cm^2^ pieces, which were then cleaned with an ultrasonic cleaner in acetone (20 min), ethanol (15 min), and deionized water (10 min). The wafer pieces were oxidized in hot Piranha solution (45 mL of H_2_SO_4_/15 mL of H_2_O_2_) for 5 min. To perform metal-assisted chemical etching (MACE), the wafer pieces were placed in a beaker containing an etchant solution made up of 5 mL of 4.6 M HF and 5 mL of 0.02 M AgNO_3_. The etching timings were 0, 5, 10, 20, 40, and 80 min. After the etching process, to remove the as-generated Ag dendrites, the substrates were immediately submerged in concentrated HNO_3_, rinsed with deionized water, and then dried at room temperature to produce the PS substrates.

### Fabrication of the SERS substrate

The deposition of Ag particles on the substrates is based on the SAM method. Colloidal Ag NPs solutions were generated at the methanol/air interface by adapting the method described in [45]. Briefly, 5 mL of the colloidal solution of Ag NPs and 5 mL of acetone were mixed in a 50 mL glass vial. This mixture was quickly poured into another glass vial containing 5 mL of hexane. The vial was shaken for 30 s and then stabilized for 10 min. The Ag NPs moved from water to hexane with the aid of acetone. The porous silicon (PS) template substrate was placed at the bottom of a beaker containing methanol. A pipette was then used to gradually transfer the Ag NPs suspension in hexane to the surface of methanol. After the hexane evaporated, a tightly packed monolayer of Ag NPs appeared at the methanol/air interface. The PS@Ag substrates were created by depositing a monolayer of Ag NPs on the substrate surface after allowing the methanol to slowly evaporate under ambient conditions.

### SERS substrate characterization

Raman spectra were obtained using a 24 mW laser and an integration time of 1000 ms. Asymmetric least-squares baseline subtraction was utilized to remove the SERS spectrum background. Each Raman spectrum represents the mean of three readings. Aqueous solutions of RhB and MLM were prepared at varying concentrations, and the SERS substrate was loaded with the analyte solution (30 µL) and air-dried at room temperature. SERS signals were then collected from random locations.

## Results and Discussion

### Fabrication of droplet-based microfluidic device using 3D printing

**Figure 1:**
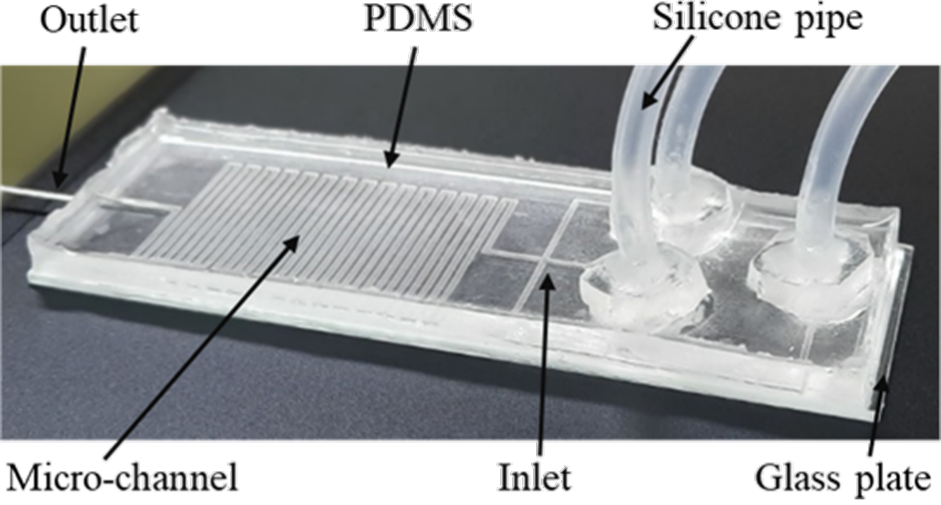
Photograph of the as-fabricated droplet-based microfluidic device.

SLA 3D printing is an additive manufacturing technology that employs a laser to transform a liquid resin into a solid plastic. When exposed to laser radiation with an appropriate wavelength, the short molecular chains in photocurable resins polymerize to form rigid or flexible solid geometries. As the printing limit of the 3D line dimension suggested by the Formlabs 3 printer manufacturer is 100 µm × 100 µm, a mold with line features was created instead of printing the microfluidic device directly. To minimize defects during the printing of the mold, the microchannel was designed with larger dimensions. Three inlets for the mixing channel were made with a width and a depth of 200 µm × 150 µm, respectively, while the mixing channel had dimensions of 400 µm × 200 µm. The total length of the serpentine-shaped microchannel, which is the reaction length, was 605.6 mm, as shown in Figure S1 (Supporting Information File 1). The photograph of the prepared microfluidic device is given in Figure 1. The droplet-based microfluidic device was constructed with three inlets for introducing reactants and oil, a long serpentine-shaped microchannel for combining chemical solutions, and one outlet for collecting suspended Ag NPs.

The PDMS cast was bound to a glass plate after peeling off from the mold. There are various surface treatment methods available, including oxygen plasma, UV/ozone, corona oxidizer, and plasma pen [46–47]. In this study, we employed a previously reported chemical treatment method that uses 1 M NaOH solution for 24 h [48]. Although this method takes longer, it is more cost-effective, straightforward, and accessible. The chemical treatment of PDMS and the glass plate aims to decorate their surfaces with silanol groups (Si–OH), enabling the two surfaces to bond chemically at the atomic level [49]. To determine the droplet size, we used two dye solutions and captured images of the observed droplets (Figure S2, Supporting Information File 1). Figures S3–S7 in Supporting Information File 1 show data on the droplet size as functions of the flow rates of aqueous solutions and oil. Our investigation revealed that the optimal flow rates are 20 and 80 µL/min for the aqueous solutions and oil, respectively.

### Synthesis of silver nanoparticles

**Figure 2:**
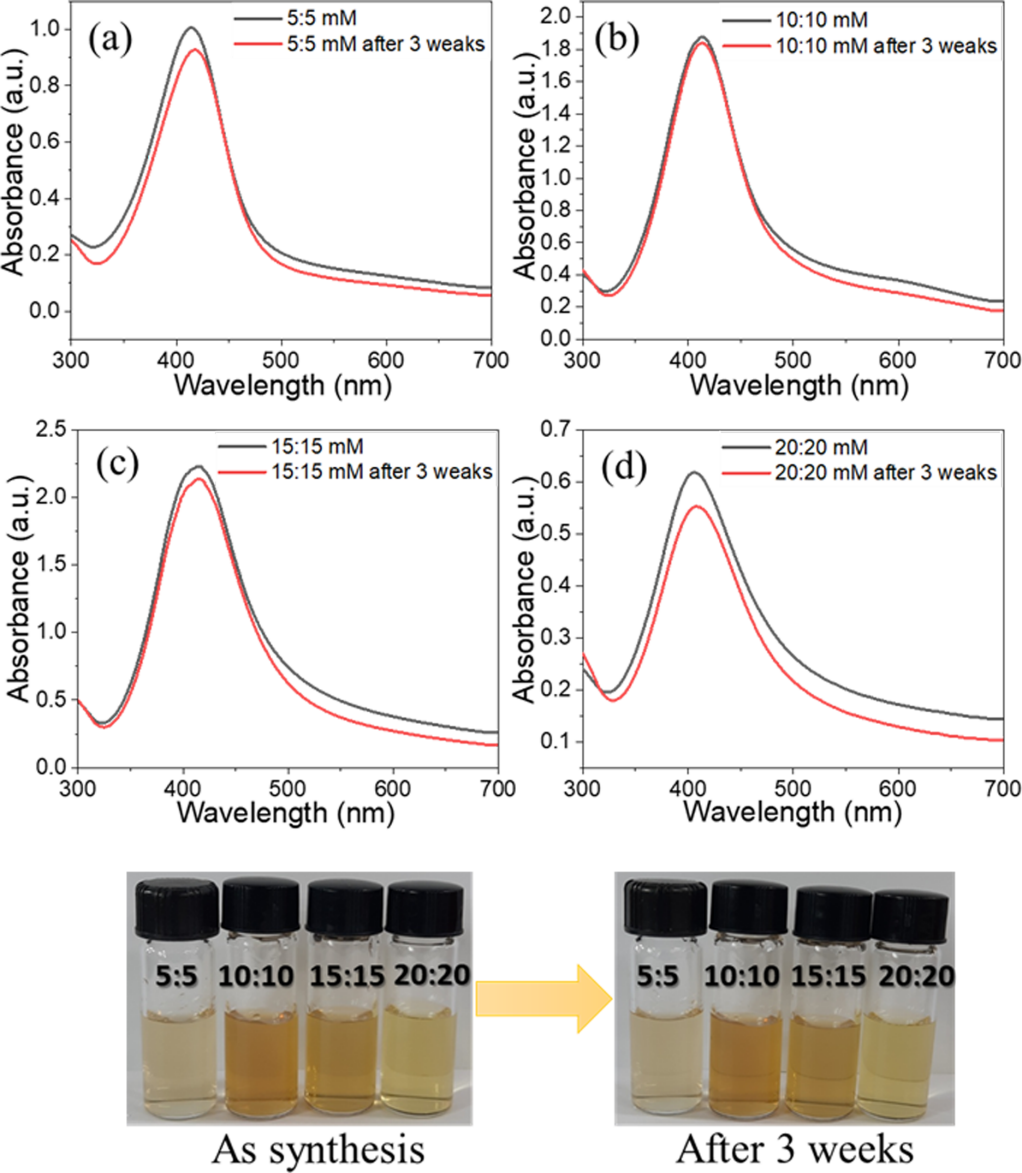
Images and absorbance spectra of Ag NPs synthesized using silver nitrate and sodium borohydride with molar ratios of (a) 5:5, (b) 10:10, (c) 15:15, and (d) 20:20 (in mM).

Different molar ratios of silver nitrate to sodium borohydride were used to produce Ag nanoparticles in the microfluidic device at room temperature, with flow rates of 20 and 80 µL/min for the aqueous solutions and oil, respectively, as described in detail in Supporting Information File 1. The use of high concentrations of AgNO_3_ resulted in the formation of numerous Ag nuclei due to a rapid reduction process. The collision frequency of these nuclei increases significantly with higher concentrations of silver nitrate, promoting the formation of larger particles. This phenomenon explains the observed color change from yellow to greenish after three weeks, as illustrated in Figure S8 (Supporting Information File 1) [50–51]. NaBH_4_ is a relatively potent reducing agent that can reduce Ag^+^. At higher concentrations of NaBH_4_, the formation of silver nanoparticles occurs more rapidly [52]. When the concentrations of silver nitrate and trisodium citrate were kept constant and the concentration of sodium borohydride was varied from 5–20 mM, the relative amount of capping agent decreased, resulting in a color change of the obtained solution from yellowish to bright yellow after three weeks, as shown in Figure S9 (Supporting Information File 1). The Ag NPs remained almost unchanged after three weeks when the ratio of silver nitrate to sodium borohydride was kept constant, especially with a molar ratio of 10:10 (in mM) (Figure 2).

**Figure 3:**
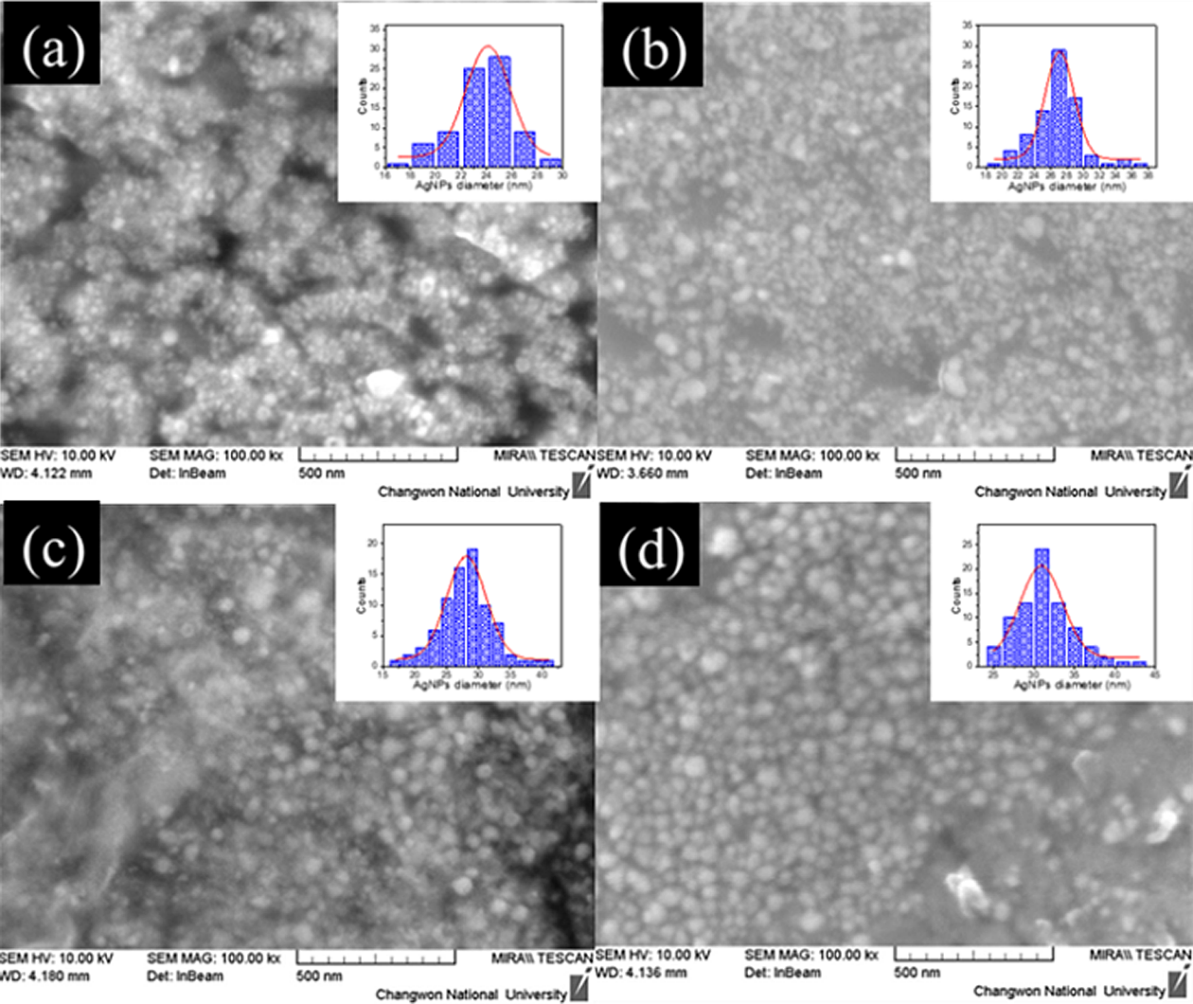
FE-SEM images of the synthesized Ag NPs with the molar ratios of AgNO_3_ and NaBH_4_ of (a) 1:1 mM, (b) 5:5 mM, (c) 10:10 mM, and (d) 15:15 mM. Insets of the figures are size distributions obtained from the ImageJ software.

FE-SEM micrographs of the samples are shown in Figure 3. The average sizes of Ag nanoparticles were estimated to be 24.10 ± 0.15, 27.07 ± 0.16, 28.07 ± 0.17, and 30.88 ± 0.31 nm by using the ImageJ program. Based on the calculated data, it is evident that the average size of Ag NPs increases with an increase in the concentration of reactants.

### Structural characterization of the SERS substrate

In Supporting Information File 1, Figure S10a shows the SEM image of the SAM of Ag NPs on the surface of methanol, while Figure S10b displays the image of the PS substrate after covering with the Ag NPs. The SEM image of PS@Ag reveals the presence of nanoscale gaps between the Ag NPs, which act as hot spots with a high electric field intensity when exposed to laser irradiation (Figure S10c). To confirm the distribution of chemical elements on the SERS substrate, energy-dispersive X-ray spectroscopy (EDS) was carried out, as illustrated in Figure S11 (Supporting Information File 1). The EDS mapping results indicate the uniformly distributed signal of the silicon wafer, while oxygen and silver are discontinuously distributed because of the space between PS and Ag NPs on the PS@Ag substrate.

### Optimization of the PS@Ag SERS substrate fabrication

To optimize the etching time of PS on the wafers, a RhB solution (10^−5^ M) was chosen for estimating the SERS signal. The wafer pieces were etched for 0, 5, 10, 20, 40, and 80 min. The corresponding substrates were denoted PS0min@Ag, PS5min@Ag, PS10min@Ag, PS20min@Ag, PS40min@Ag, and PS80min@Ag, respectively. The SEM images revealed that the SAM of Ag NPs on the substrate without etching is not uniform, as shown in Figure S12 (Supporting Information File 1). The SAMs of the substrates with etching times of 5 and 10 min exhibited some islands that were not continuous, as shown in Figure S12b,c (Supporting Information File 1). When the etching time was extended to 20 and up to 80 min, the silver nanoparticles were well ordered, especially after an etching time of 40 min (Figure S12d–f, Supporting Information File 1).

**Figure 4:**
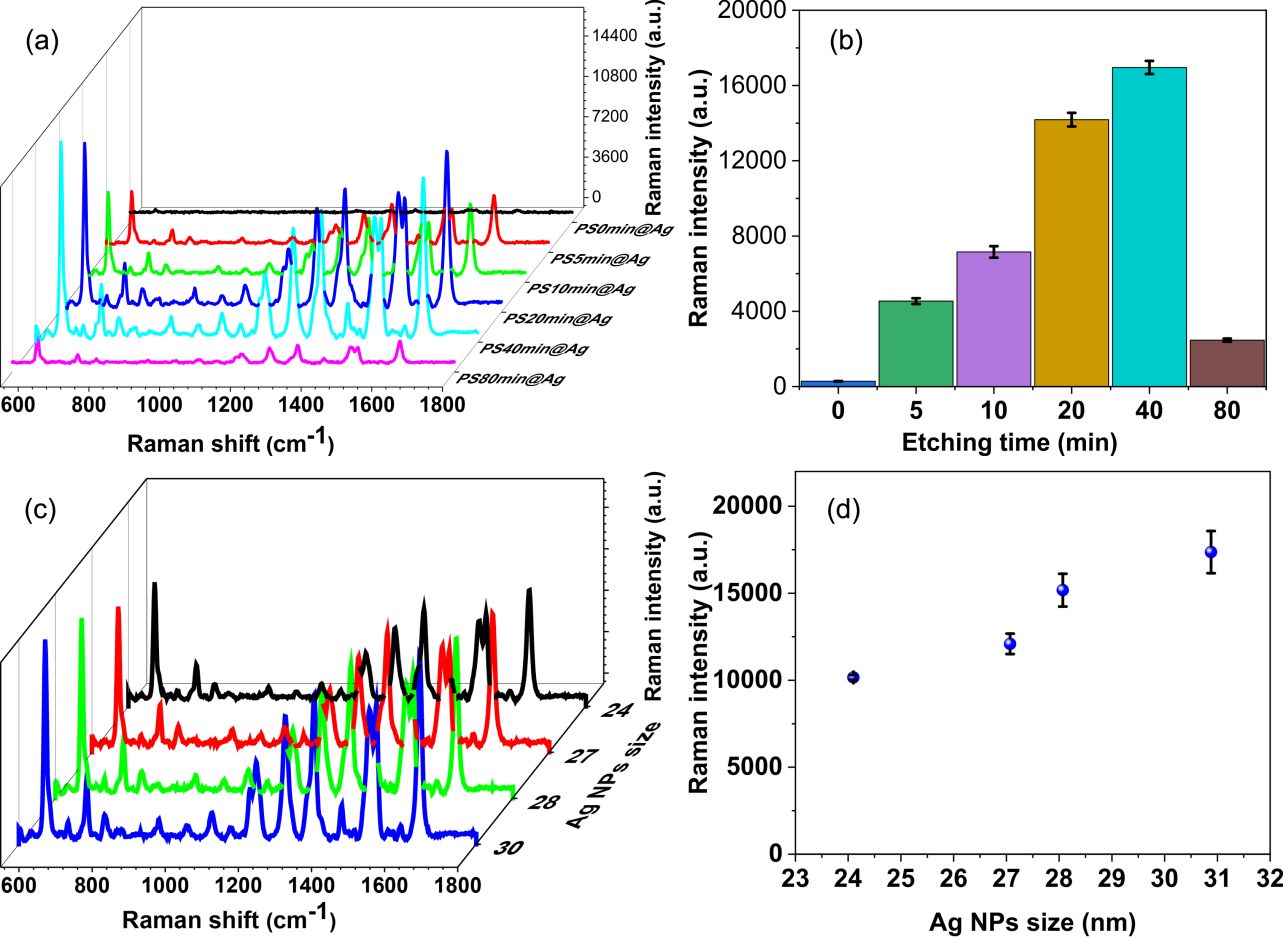
(a) SERS spectra based as a function of the PS etching time. (b) Intensity of the 621 cm^−1^ SERS speak as a function of the PS etching time. (c) SERS spectra of RhB on Ag NPs with different diameters on the PS40min@Ag substrate. (d) Intensity of the 621cm^−1^ SERS peak as a function of the size of the Ag NPs.

Figure 4a shows the SERS spectra of RhB on substrates with different etching times. The spectra exhibit peaks at 621, 1199, 1279, 1358, 1508, 1528, and 1647 cm^−1^. The peaks at 621, 1199, and 1279 cm^−1^ are related to deformation vibrations of the xanthene ring, the stretching of the C–C bridge bands, and the bending of the aromatic C–H bonds, respectively. The peaks at 1358, 1508, and 1528 cm^−1^ are linked to the aromatic C–C bending, while the peak at 1647 cm^−1^ is determined by the bending and stretching of the C–C bonds [53].

The SERS intensity increases with an extension of the etching time of the PS substrate from 0 to 40 min, and it decreases quickly when longer etching times are applied. The longer etching results in the expansion of pores in the silicon (100) wafer. These pores retain the Ag particles. As the pores become deeper and larger, the distances between the Ag particles increase, thereby reducing the hot spot effect on the Raman signal. The intensities of the peaks from the sample PS0min@Ag are significantly lower than those of the other samples. In comparison with the PS40min@Ag sample, the intensity of the peak at 621 cm^−1^ is approximately sixty times lower (Figure 4b). This confirms that the etching time of PS is a critical parameter for the fabrication of the SERS substrate. The substrate with an etching time of 40 min (PS40min@Ag) exhibited the best SERS performance and thus was chosen as the optimal substrate for the subsequent tests.

The influence of the Ag NP size on the SERS signals was studied. Four different sizes of Ag NPs (24, 27, 28, and 30 nm) were self-assembled on the PS40min@Ag substrate, and the SERS spectra of RhB (10^−5^ M) were collected, as shown in Figure 4c. A slight increase in intensity was observed as the diameter of Ag NPs increased from 24 to 30 nm (Figure 4d). This is because the gaps between the Ag particles become smaller when the size of the particles increases, resulting in a stronger hot spot effect. However, when the particle size exceeds a critical threshold, the Ag particles come into contact with each other, causing the hot spots to dissipate. Based on these findings, we selected Ag NPs with a diameter of 28 nm as the optimal choice for the SERS substrate and used them for subsequent experiments.

### Relationship between RhB concentration and Raman intensity

**Figure 5:**
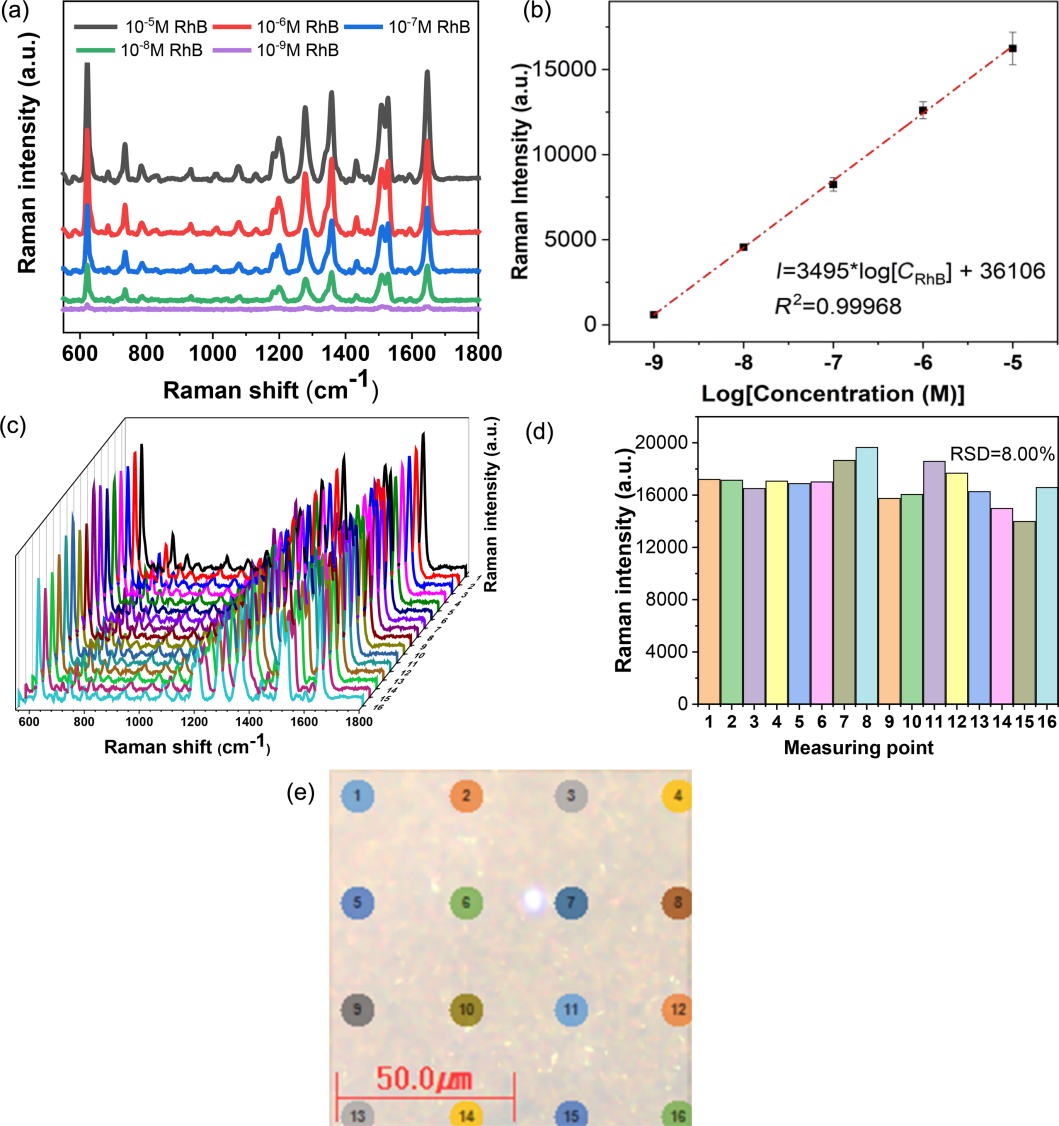
(a) Raman spectra of RhB solutions in the range from 10^−9^ to 10^−5^ M. (b) Intensity of the Raman peak at 621 cm^−1^ as a function of the RhB concentration. (c) SERS spectra and (d) SERS intensity of the 621 cm^−1^ peak at 16 positions of the SERS substrate, which are given in (e).

The SERS spectra of RhB samples with different concentrations, ranging from 10^−9^ to 10^−5^ M, were collected using the PS40min@Ag substrate (Figure 5a). The SERS intensity of RhB increased as the concentration increased. Figure 5b illustrates the correlation between RhB concentration and the peak intensity at 621 cm^−1^, which was used to create a linear calibration with a high regression coefficient of *R*^2^ = 0.99968. By applying the 3σ/*s* method, where σ is the standard deviation of the blank and *s* is the slope of the linear regression equation, the limit of detection was found to be 1.94 × 10^−10^ M. The SERS substrates showed an enhancement factor (EF) of 8.59 × 10^6^ for the 621 cm^−1^ peak at an RhB concentration of 10^−9^ M. The calculation details for the EF can be found in the “Enhancement factor calculation” section of Supporting Information File 1.

To investigate the uniformity of the SERS substrate, Raman mapping was performed at 16 measuring positions in an area of 100 µm × 100 µm . RhB with a concentration of 10^−5^ M was added to the PS40min@Ag substrate. Figure 5c displays the SERS spectra collected from each position, while Figure 5d shows the SERS peak intensity at 621 cm^−1^ for the different positions. The relative standard deviation (RSD) was calculated to be 8%, indicating that the PS@Ag structure yielded homogeneous results because of the even deposition of the self-assembled monolayer of Ag NPs on the PS substrate.

### Detection of melamine using PS@Ag SERS substrate

**Figure 6:**
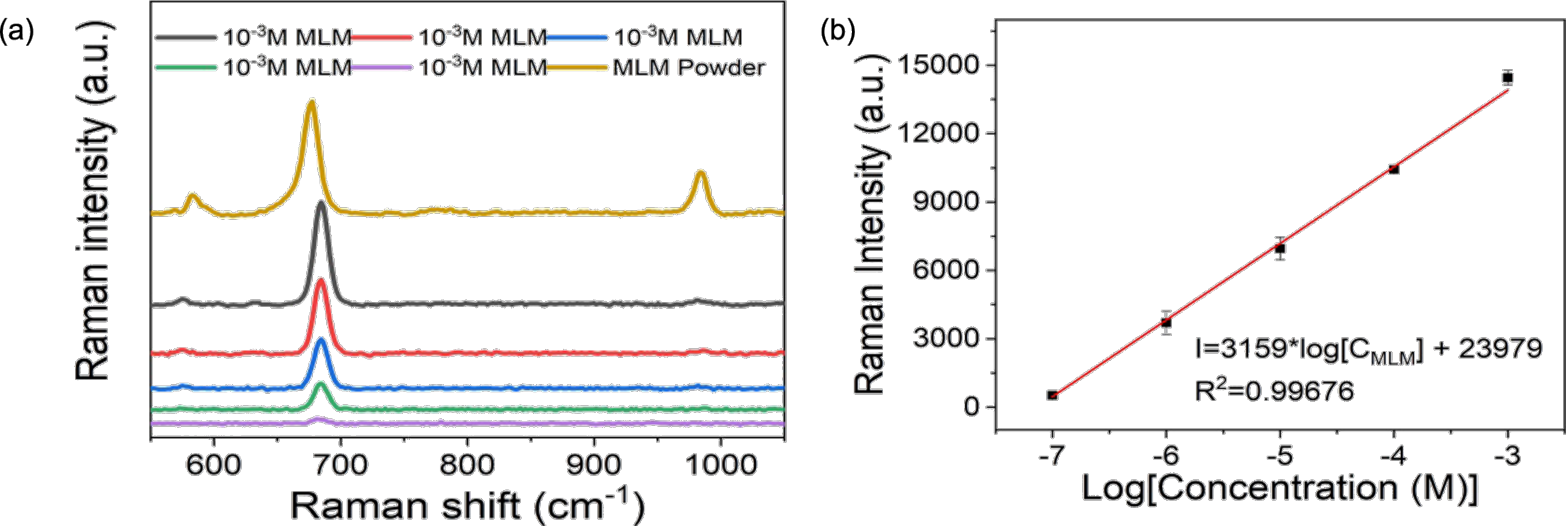
(a) Raman spectra of powder and trace concentrations of MLM (10^−7^ to 10^−3^ M). (b) SERS intensity of the 682 cm^−1^ peak as a function of the MLM concentration.

SERS spectra of MLM solutions at different concentrations (10^−7^ to 10^−3^ M) on the PS@Ag substrates were recorded, as shown in Figure 6a. The Raman spectrum of MLM exhibits strong peaks at 583, 676, and 983 cm^−1^. The peak at 583 cm^−1^ is related to a mixed mode of N–C–N bending and NH_2_ twisting vibrations. The peak at 676 cm^−1^ is attributed to the plane deformation modes of the triazine ring, and the peak at 983 cm^−1^ is ascribed to C–N–C bending vibrations [54–55]. The Raman spectra of MLM on the PS@Ag SERS substrate shows peaks at 585, 679, and 985 cm^−1^, which are shifted compared to those in the Raman spectrum of MLM because of the interaction between MLM and the Ag surface.

The SERS intensity at the fingerprint peak of 682 cm^−1^ as a function of the MLM concentration is displayed in Figure 6b. The calculated limit of detection for MLM was found to be 2.8 × 10^−8^ M, which is significantly below the safety limit established by the US Food and Drug Administration [56]. The SERS substrates displayed an impressive EF of 8.21 × 10^3^ at the 682 cm^−1^ peak for an MLM concentration of 10^−7^ M. Further information on the calculation of the EF can be found in the “Enhancement factor calculation” section of Supporting Information File 1.

## Conclusion

Using 3D printing, a droplet-based microfluidic device was successfully fabricated without the need for expensive and time-consuming photolithography. The resulting devices were utilized to produce uniformly distributed silver nanoparticles that were then applied to the surface of a porous silicon substrate via a self-assembly technique. This created SERS substrates detecting RhB and MLM with detection limits of 1.94 × 10^−10^ and 2.8 × 10^−8^ M, respectively. Enhancement factors of 8.59 × 10^6^ and 8.21 × 10^3^ were achieved for RhB and MLM, respectively. SERS mapping showed the substrate to have good homogeneity with a relative standard deviation of the peak intensities of 8%. These results demonstrate the excellent analytical performance of the PS@Ag SERS substrate, making it a promising tool for detecting environmental pollutants and ensuring food safety.

## Supporting Information

The Supporting Information features the following: four steps of the fabrication process of a microfluidic device; design of the mold; optimization of droplet generation; synthesis of silver nanoparticles in the droplet-based microfluidic device; FESEM images and EDX spectra of the SERS substrate; SEM images of the SERS substrate with RhB; and calculation of the enhancement factors for RhB and MLM.

File 1 Additional experimental data.
