# Supplementary material for: Silver-based SERS substrates fabricated using a 3D printed microfluidic device
Source: Beilstein J Nanotechnol. 2023 Jul 21;14:793–803. doi: 10.3762/bjnano.14.65 (PMC10366445; doi:10.3762/bjnano.14.65)
Supplement: File 1 — Additional experimental data. [file Beilstein_J_Nanotechnol-14-793-s001.pdf]

BEILSTEIN JOURNAL OF NANOTECHNOLOGY

Supporting Information

for

Silver-based SERS substrates fabricated using a 3D printed microfluidic device

Phommachith Sonexai, Minh Van Nguyen, Bui The Huy and Yong-Ill Lee

Beilstein J. Nanotechnol. **2023,** *14,* 793–803. doi:10.3762/bjnano.14.65

Additional experimental data

License and Terms: This is a supporting information file under the terms of the Creative Commons Attribution License (https://creativecommons.org/licenses/by/4.0). Please note that the reuse, redistribution and reproduction in particular requires that the author(s) and source are credited and that individual graphics may be subject to special legal provisions.
The license is subject to the *Beilstein Journal of Nanotechnology* terms and conditions: (https://www.beilstein-journals.org/bjnano/terms)

1. Fabrication of droplet-based microfluidic device using 3D printing

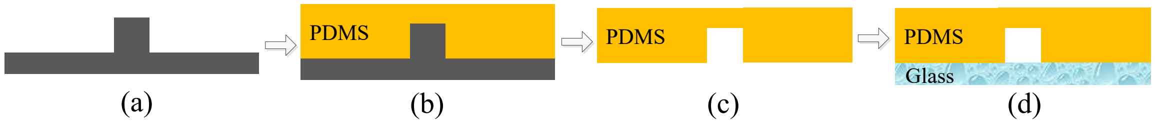

Scheme S1: Four steps of the fabrication process of a microfluidic device: (a) 3D printing of the mold; (b) PDMS casting; (c) PDMS peel-off; and (d) PDMS–glass bonding.

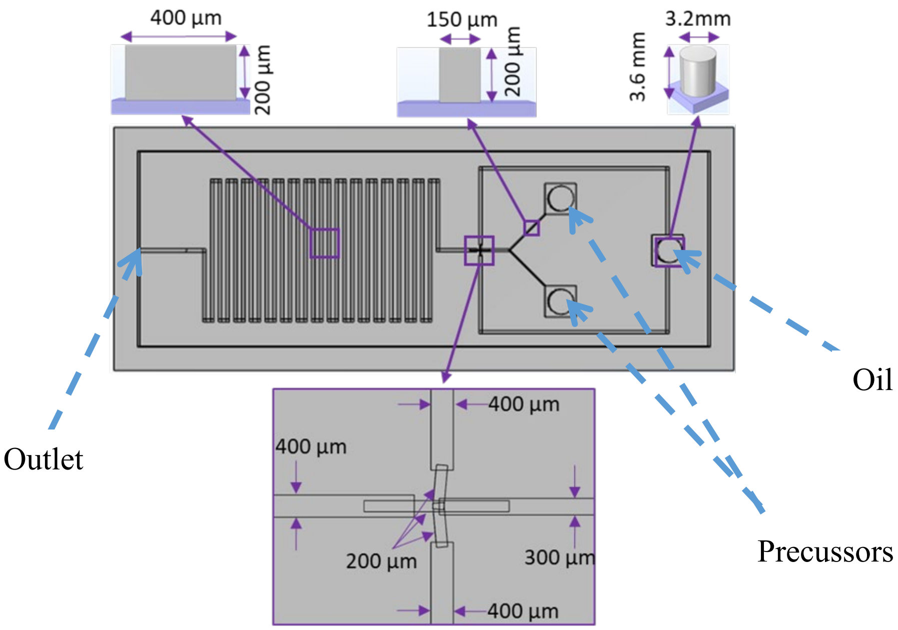

Figure S2: Design of the mold with a serpentine shape by Solidworks Professional 2022 SP3.1 software.

2. Optimization of droplet generation

To determine the size of the droplet, we applied two colors of fluid and took photos at three spots as illustrated in Figure S2.

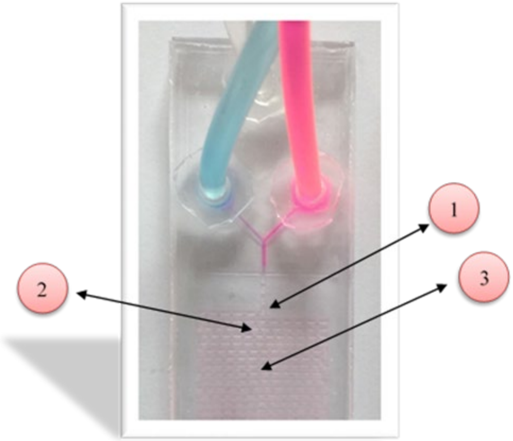

Figure S2: Droplet-based microfluidic device.

The droplet-based microfluidic device consists of three inlets: one for oil and two inlets for the two precursors, as shown in Figure S1. The red and blue channels represent the colors of the aqueous solutions used in the visual experiments. In this work, the two precursors are $AgNO_3$ and $NaBH_4$ solutions. The aqueous channels containing these precursors react before reaching the intersection. At the intersection, the flow of Ag particles in water is separated by the presence of oil. The channel segment containing a mixture of reactive precursors was designed to be short enough so that the mixing time has a small effect on the generation of the initial seeds before the solution droplets are formed. If the segment's length is too short, the oil flow may push back the reactants, which affects droplet generation. The growth of the AgNPs occurs in the microdroplets. The length of the microchannel was designed long enough so that the reaction time of the reactants in the microdroplets was smaller than the living time of the microdroplets in the channel to ensure complete chemical reaction.

The sizes of droplets based on the flow rate ratio between oil and solutions were investigated. At a ratio of 5:5 µL/min (aqueous solutions/oil), the size of the droplets could not be determined because the camera’s angle was smaller than the droplet size (Figure S3). Therefore, we continued to increase the flow rate of oil from 5 µL/min to 20 µL/min and to 40 µL/min such that the size of

the droplets could be determined. However, the droplets that generate under these conditions are not stable. Hence, to improve the stability of droplets, we have increased the flow rate ratio of aqueous solutions and oil from 5 μL/min to 10 μL/min and to 20 μL/min. Uniform and stable droplets were obtained, as displayed in Figure S4 and Figure S5. As illustrated in Figure S6, the size of the droplets is inversely proportional to the flow rate. It is worth noting that droplets generated at a lower flow rate of solutions (5 μL/min and 10 μL/min) were not stable. Stable droplets were formed when the flow rate of the aqueous solution was increased to 20 μL/min, notably at the flow rate of 1:3.5 and 1:4 (aqueous solutions/oil). Therefore, all silver nanoparticles in this article were synthesized with a flow rate ratio of aqueous solution to oil of 1:4 (20:80 μL/min).

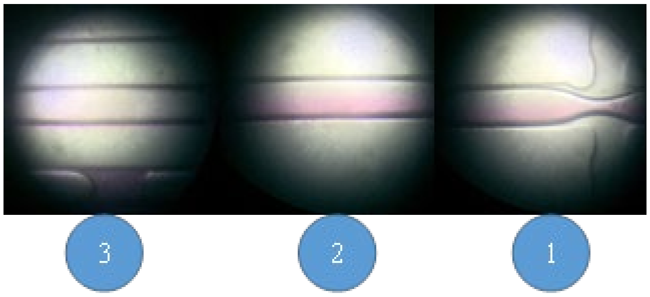

Figure S3: Droplet size distribution while maintaining the flow rate of aqueous solutions at 5 μL/min and changing the oil's flow rate from 5.5 μL/min (1), to 5.2 μL/min (2) and to 5.4 μL/min (3).

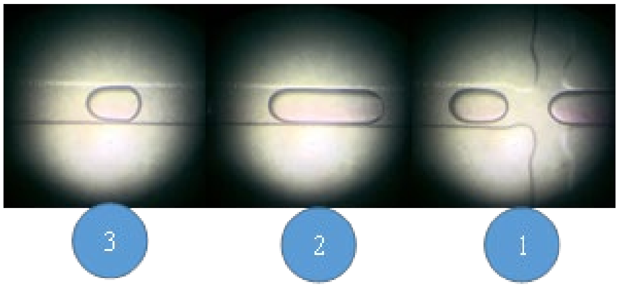

Figure S4: Droplet size distribution while maintaining the flow rate of solutions at 5 µL/min and changing the oil's flow rates from 5 µL/min (1) to 20 µL/min (2) and to 40 µL/min (3).

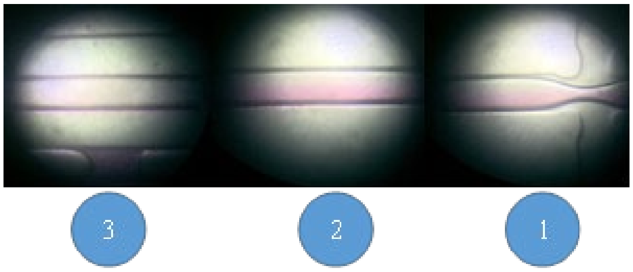

Figure S3: Droplet size distribution while maintaining the flow rate of aqueous solutions at 10 µL/min and changing the oil's flow rates from 10 µL/min(1) to 30 µL/min (2) and to 60 µL/min (3).

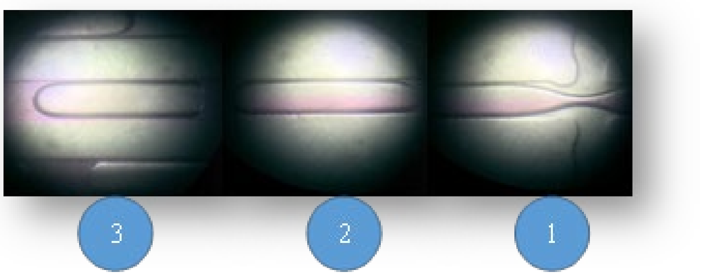

Figure S6: Droplet size distribution while maintaining the flow rate of solutions at 20 µL/min and changing the oil's flow rates from 20 µL/min (1) to 40 µL/min (2) and to 80 µL/min (3).

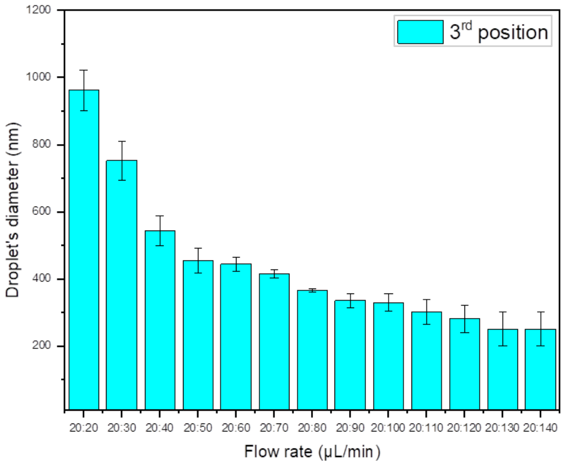

Figure S7: Droplet diameter distribution measured at the third position of the microfluidic chip obtained at a flow rate of solutions at 20 μL/min and changing the oil's flow rate from 20 to 140 μL/min.

3. Synthesis of silver nanoparticles in the droplet-based microfluidic device

Through chemical reduction, Ag NPs were synthesized using the microfluidic device. The Ag NPs were synthesized from silver nitrate ($AgNO_3$) as an Ag^+ source, sodium citrate (TSC) as a stabilizer, and sodium borohydride ($NaBH_4$) as a reducing agent [1,2]. Ag NPs were synthesized at room temperature with a flow rate ratio of aqueous solutions and oil of 20 and 80 μL/min, respectively, with various molar ratios of silver nitrate to sodium borohydride, as shown in Figure S8 and Figure S9.

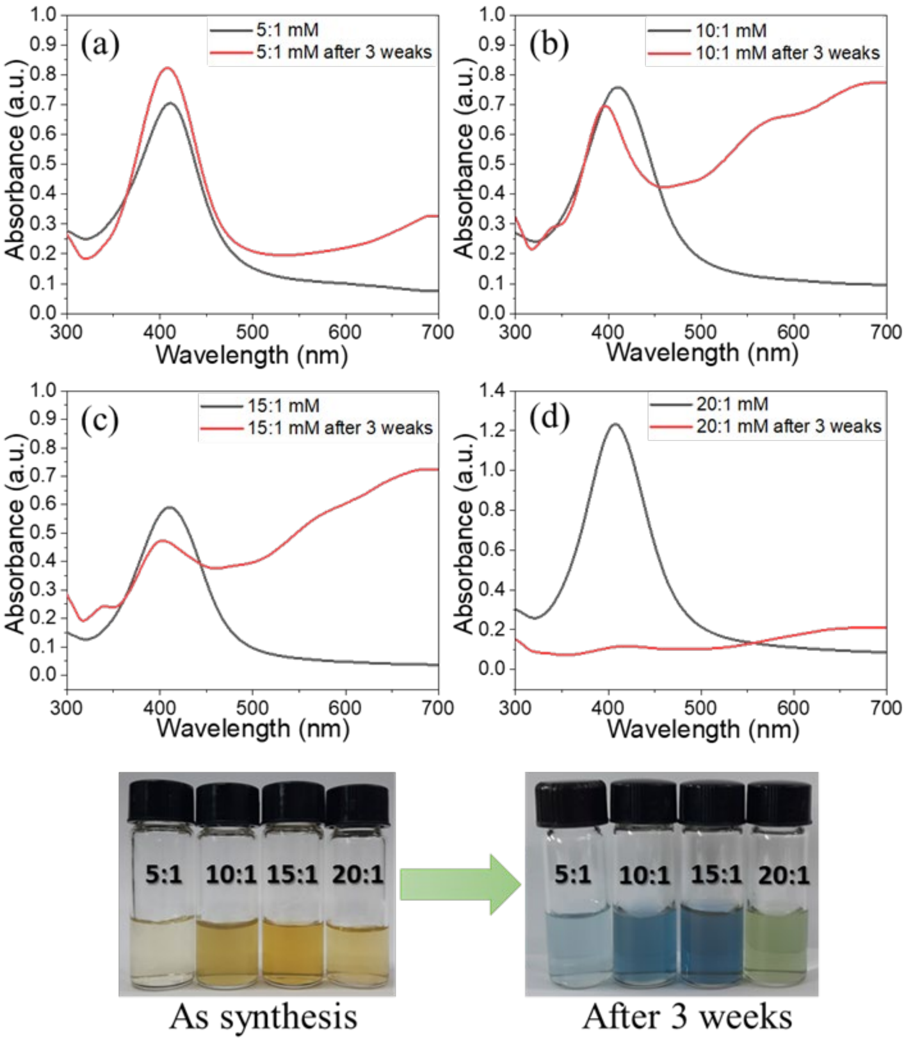

Figure S8: Optical images and absorbance spectra of Ag NPs synthesized using silver nitrate and sodium borohydride: 5:1 mM (a), 10:1 mM (b), 15:1mM (c), and 20:1 mM (d).

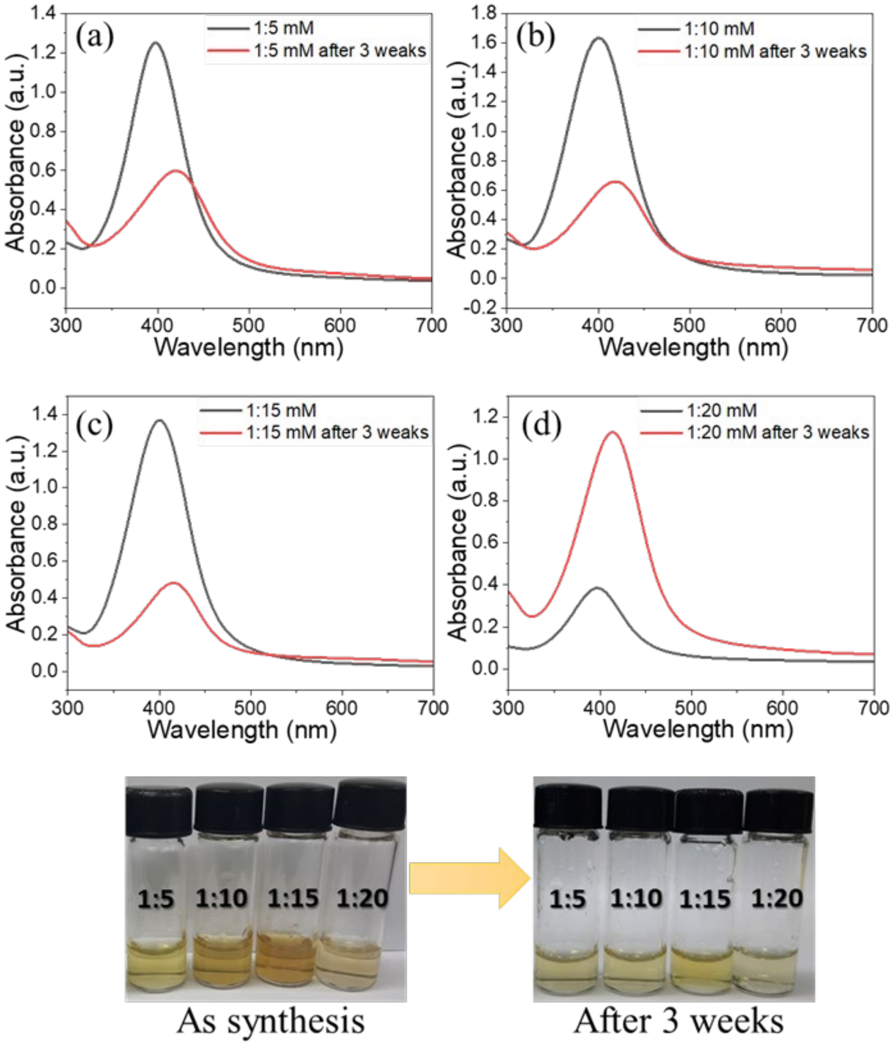

Figure S9: Optical images and absorbance spectra of Ag NPs synthesized using silver nitrate and sodium borohydride: 1:5 mM (a), 1:10 mM (b), 1:15 mM (c), 1:20 mM (d).

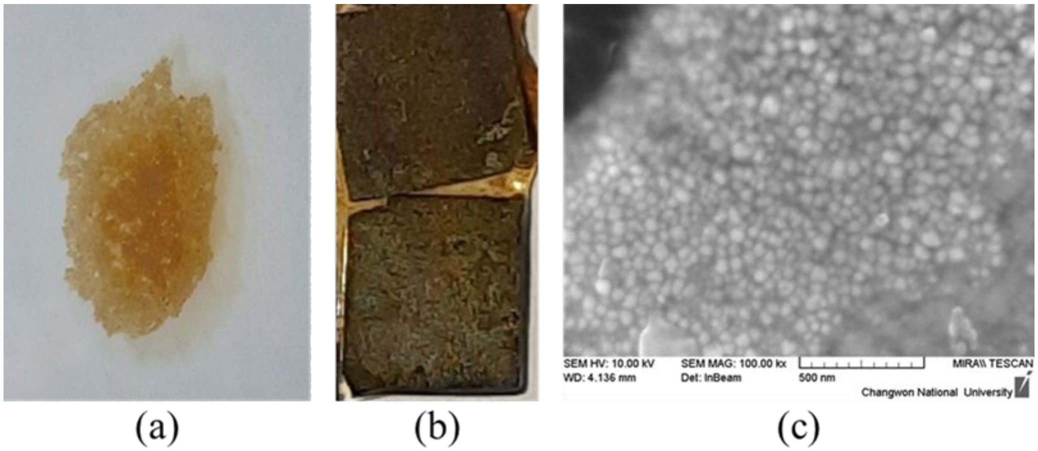

Figure S10: (a) SAM of Ag NPs on the surface of methanol, (b) PS@Ag, (c) FE-SEM image of PS@Ag.

4. FESEM image and EDX spectra of the SERS substrate

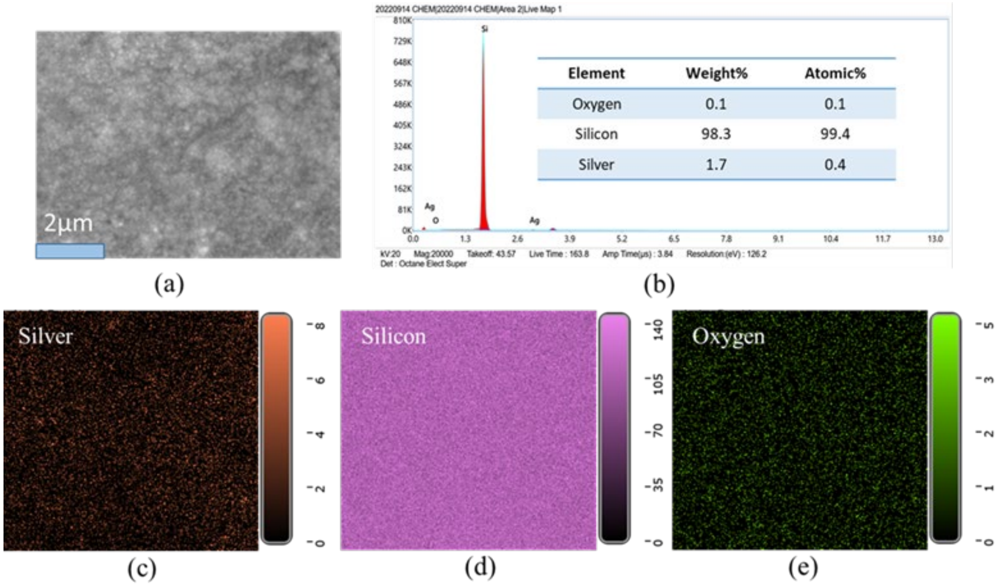

Figure S11: (a) FE-SEM image of the PS@Ag substrate surface. (b) EDX spectrum with the inset table listing the weight and atomic concentrations of oxygen, silicon, and silver. Elemental mapping by EDX of silver (c), silicon (d), and oxygen (e).

5. SEM images of the SERS substrate with RhB

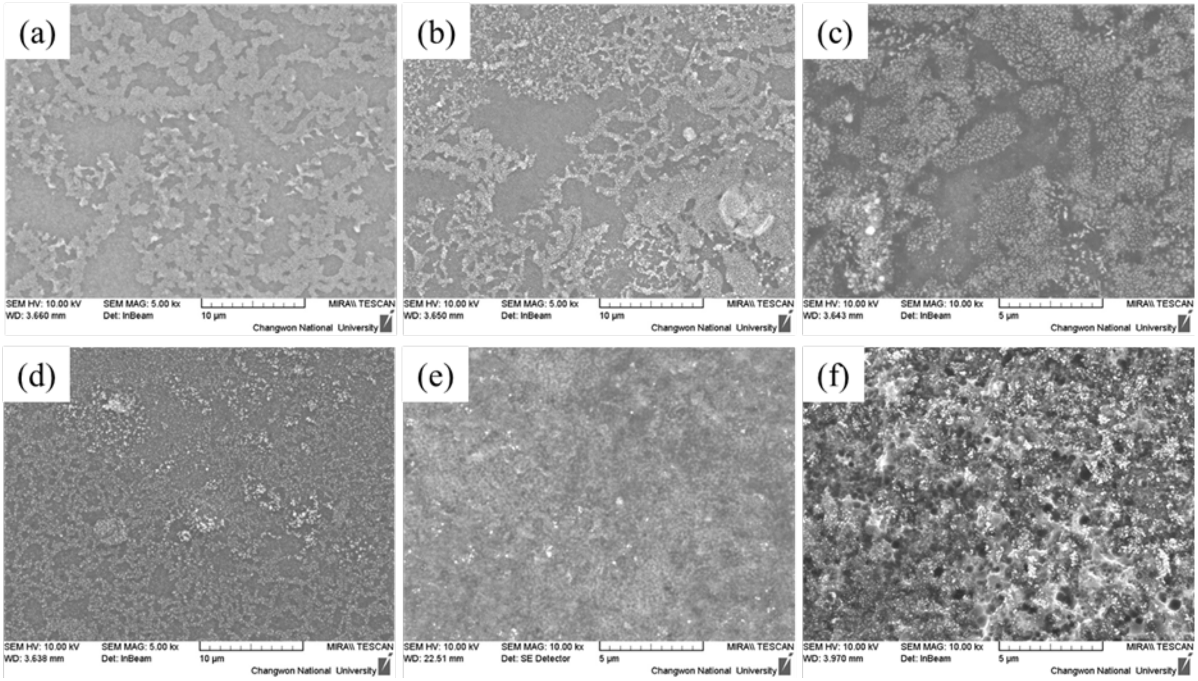

Figure S12: SERS substrate after different etching times: (a) PS0min@Ag, (b) PS5min@Ag, (c) PS10min@Ag, (d) PS20min@Ag, (e) PS40min@Ag, and (f) PS80min@Ag.

6. Calculating the enhancement factor for RhB

The EF of the SERS substrates at the 621 cm^{-1} peak at a RhB concentration of 10^{-9} M was calculated to be 8.59×10^{6}. Taking into consideration the strong fluorescence of rhodamine B in the solid state, SERS intensity is compared with normal Raman intensity from the molecules on a blank silicon substrate under laser irradiation. The EF calculation is given in Equation 1 [3]:

$$\mathrm{EF} = \frac{I_{\mathrm{SERS}}}{I_{\mathrm{R}}}.\frac{N_{\mathrm{R}}}{N_{\mathrm{SERS}}} \quad (1)$$

$$N_{\mathrm{R}} = C_{\mathrm{R}}.V_{\mathrm{R}}.N_{\mathrm{A}}.\frac{S_{\mathrm{laser}}}{S_{\mathrm{R}}} \quad (2)$$

$$N_{\mathrm{SERS}} = C_{\mathrm{SERS}}.V_{\mathrm{SERS}}.N_{\mathrm{A}}.\frac{S_{\mathrm{laser}}}{S_{\mathrm{SERS}}} \quad (3)$$

Substituting Equation 3 and Equation 4 into Equation 2 yields:

$$\mathrm{EF} = \frac{I_{\mathrm{SERS}}}{I_{\mathrm{R}}}.\frac{C_{\mathrm{R}}.V_{\mathrm{R}}.S_{\mathrm{SERS}}}{C_{\mathrm{SERS}}.V_{\mathrm{SERS}}.S_{\mathrm{R}}} \quad (4)$$

where I_{R}, C_{R}, V_{R}, and S_{R} are the Raman intensity, concentration, volume, and surface area of the RhB solution dripped on the blank silicon substrate, respectively. I_{SERS}, C_{SERS}, V_{SERS}, and S_{SERS} are the SERS intensity, concentration, volume, and surface area of the RhB solution dripped on the

SERS substrate, respectively.

$V_R = V_{SERS} = 30\ \mu L$; $S_R = S_{SERS} = 0.5 \times 0.5\ cm^2$

$I_{SERS} = 656.291$ at 621 cm^{-1} peak; $C_{SERS} = 10^{-9}$ M

$I_R = 763.245$ at 621 cm^{-1} peak; $C_R = 0.01$ mol/L

From Equation 5, the EF of RhB at 621 cm^{-1} on the SERS substrate was calculated to be 8.59×10^6.

7. Calculating the enhancement factor for MLM

The SERS peak at 682 cm^{-1} was chosen to calculate the enhancement factor of the PS@Ag SERS substrate with MLM at a concentration of 10^{-7} M. The EF can be calculated from Equation 5, in which the SERS intensity is compared with the normal Raman intensity from MLM powder [4].

$$\mathrm{EF} = \left(\frac{I_{\mathrm{SERS}}}{I_{\mathrm{R}}}\right) . \left(\frac{N_{\mathrm{bulk}}}{N_{\mathrm{SERS}}}\right) \tag{5}$$

where I_{SERS} is the intensity of the selected vibrational mode of the adsorbed MLM molecules on the SERS substrate. $I_{SERS} = 623.297$ at the Raman peak of 682 cm^{-1} at a MLM concentration of 10^{-7} M. $I_{bulk} = 15772.544$ at 682 cm^{-1}. I_R is the Raman intensity of MLM powder at 682 cm^{-1}. N_{bulk} is the number of molecules in the volume of MLM powder irradiated by the laser. N_{SERS} is the number of MLM molecules under the area covered by the incident laser spot. Assuming that the molecules are distributed regularly on the substrate surface, N_{SERS} is calculated by Equation 6:

$$N_{\mathrm{SERS}} = C \cdot V \cdot N_{\mathrm{A}} . \frac{S_{\mathrm{laser}}}{S_{\mathrm{SERS}}} \tag{6}$$

where $C = 10^{-7}$ M is the concentration of MLM chosen for EF calculation, and $V = 30\ \mu L$, is the total volume of the MLM solution dripped on the SERS substrate. S_{SERS} is the SERS substrate surface area ($0.5 \times 0.5\ cm^2$). $N_A = 6.022 \times 10^{23}$ is the Avogadro number. $S_{laser} = \pi d^2/4$ is the area of the laser spot on the substrate surface, with $d = 1.22 \frac{\lambda}{\mathrm{NA}}$, where NA = 0.45 (for a 20× objective lens and a working distance of 3.1 mm), $\lambda = 633$ nm. Then, $d = 1.7\ \mu m$. The total weight of MLM molecules in the cylinder with height H and diameter d is calculated by the following formula: $m = \left(\frac{\pi}{4}\right) \cdot d^2 \cdot H \cdot \rho$, where ρ is the weight density. The number of MLM molecules in the cylinder is calculated using Equation 7:

$$N_{\mathrm{bulk}} = \frac{\left(\frac{\pi}{4}\right) \cdot d^2 \cdot H \cdot \rho \cdot N_{\mathrm{A}}}{M} \tag{7}$$

where H = 2 μm is the penetration depth of the laser. ρ = 1.573 g/cm^3 is the density of MLM powder. M = 126.123 g/mol is the molecular weight of MLM. From Equation 5, the EF of MLM on the PS@Ag SERS substrate was calculated to be 8.21 × 10^3.

References

1. Hao, N.; Nie, Y.; Xu, Z.; Zhang, J. X. J. *J. Colloid Interface Sci.* **2019,** *542,* 370–378. doi:10.1016/j.jcis.2019.02.021
2. Sarjuna, K. *ECS Trans.* **2022,** *107,* 4113. doi:10.1149/10701.4113ecst
3. Zhang, L.; Li, P.; Luo, L.; Bu, X.; Wang, X.; Zhao, B.; Tian, Y. *Appl. Spectrosc.* **2017,** *71,* 2395–2403. doi:10.1177/0003702817711700
4. Phatangare, A. B.; Dhole, S. D.; Dahiwale, S. S.; Bhoraskar, V. N. *Appl. Surf. Sci.* **2018,** *441,* 744–753. doi:10.1016/j.apsusc.2018.02.057
